# Construction of a Full-Length Transcriptome of Western Honeybee Midgut Tissue and Improved Genome Annotation

**DOI:** 10.3390/genes15060728

**Published:** 2024-06-01

**Authors:** He Zang, Sijia Guo, Shunan Dong, Yuxuan Song, Kunze Li, Xiaoxue Fan, Jianfeng Qiu, Yidi Zheng, Haibin Jiang, Ying Wu, Yang Lü, Dafu Chen, Rui Guo

**Affiliations:** 1 College of Bee Science and Biomedicine, Fujian Agriculture and Forestry University, Fuzhou 350002, China; zanghe321@163.com (H.Z.); guosijia1998@163.com (S.G.); 13859090784@163.com (S.D.); 15757057565@163.com (Y.S.); kunze0515@163.com (K.L.); imfanxx@163.com (X.F.); jfqiu@fafu.edu.cn (J.Q.); zzz974429@163.com (Y.Z.); dfchen826@fafu.edu.cn (D.C.); 2 National & Local United Engineering Laboratory of Natural Biotoxin, Fuzhou 350002, China; 3 Apitherapy Research Institute of Fujian Province, Fuzhou 350002, China; 4 Apiculture Science Institute of Jilin Province, Jilin 132000, China; jhb18047513706@163.com (H.J.); wy569703@163.com (Y.W.); 5 Mudanjiang Branch of Heilongjiang Academy of Agricultural Sciences, Mudanjiang 157000, China; 6220623004@fafu.edu.cn

**Keywords:** *Apis mellifera*, full-length transcriptome, third-generation sequencing, nanopore sequencing, reference genome

## Abstract

Honeybees are an indispensable pollinator in nature with pivotal ecological, economic, and scientific value. However, a full-length transcriptome for *Apis mellifera*, assembled with the advanced third-generation nanopore sequencing technology, has yet to be reported. Here, nanopore sequencing of the midgut tissues of uninoculated and *Nosema ceranae*-inoculated *A. mellifera* workers was conducted, and the full-length transcriptome was then constructed and annotated based on high-quality long reads. Next followed improvement of sequences and annotations of the current reference genome of *A. mellifera.* A total of 5,942,745 and 6,664,923 raw reads were produced from midguts of workers at 7 days post-inoculation (dpi) with *N. ceranae* and 10 dpi, while 7,100,161 and 6,506,665 raw reads were generated from the midguts of corresponding uninoculated workers. After strict quality control, 6,928,170, 6,353,066, 5,745,048, and 6,416,987 clean reads were obtained, with a length distribution ranging from 1 kb to 10 kb. Additionally, 16,824, 17,708, 15,744, and 18,246 full-length transcripts were respectively detected, including 28,019 nonredundant ones. Among these, 43,666, 30,945, 41,771, 26,442, and 24,532 full-length transcripts could be annotated to the Nr, KOG, eggNOG, GO, and KEGG databases, respectively. Additionally, 501 novel genes (20,326 novel transcripts) were identified for the first time, among which 401 (20,255), 193 (13,365), 414 (19,186), 228 (12,093), and 202 (11,703) were respectively annotated to each of the aforementioned five databases. The expression and sequences of three randomly selected novel transcripts were confirmed by RT-PCR and Sanger sequencing. The 5′ UTR of 2082 genes, the 3′ UTR of 2029 genes, and both the 5′ and 3′ UTRs of 730 genes were extended. Moreover, 17,345 SSRs, 14,789 complete ORFs, 1224 long non-coding RNAs (lncRNAs), and 650 transcription factors (TFs) from 37 families were detected. Findings from this work not only refine the annotation of the *A. mellifera* reference genome, but also provide a valuable resource and basis for relevant molecular and -omics studies.

## 1. Introduction

Honeybees, which are recognized as social insects, play a pivotal part in pollination for up to 70% of crop species and wild plants worldwide [1,2]. Consequently, they are of significant importance to agricultural economics, food security, environmental ecology, and scientific research. Given their gentle nature, their strong foraging and productivity capacities, and the ease with which large colonies can be maintained, the western honeybee (*Apis mellifera*) enjoys global favor [3,4].

Third-generation sequencing technologies, commonly referred to as long-read sequencing technologies, enable the direct sequencing of large DNA fragments. This offers significant advantages in de novo genome assembly and metagenomics [5]. Nanopore sequencing technology, as one of the leading third-generation sequencing technologies, is capable of generating reads up to 100,000 bases in length [6] and thereby has substantial advantages in the identification of full-length transcripts. A full-length transcriptome is beneficial for performing molecular studies in organisms ranging from the identification of alternative splicing (AS) and alternative polyadenylation (APA) to the precise quantification of genes and transcripts, especially when there is no reference genome available for the organism [7,8,9,10]. Nanopore sequencing has now provided full-length transcriptomes of animals, plants, and microorganisms such as Muscovy ducklings (*Cairina Moschata*) [11], *Asparagus* [12], and *Saccharomyces cerevisiae* [13]. In insects, the full-length transcriptomes of species such as *Cydia pomonella* L. [14] and *Bactrocera dorsalis* [15] have been reported. Separately, the full-length transcriptome of the plant *Fraxinus chinensis* [16] was also studied.

Second-generation sequencing technology has been widely applied in dissecting many aspects of honeybees, such as genetics [17], ethology [18], and host-pathogen interaction [19]. For instance, following deep sequencing utilizing the Illumina platform, Manfredini et al. analyzed the change in gene-expression patterns in brains of *A. mellifera* queens from virgin to mated reproductive status and discovered that the mating process significantly altered the expression of genes related to vision, chemoreception, metabolism, and immunity [18]. Comparatively, third-generation-sequencing-based studies on honeybees are currently very limited. Recently, Zheng et al. [20] reported the first full-length transcriptome of *A. mellifera* based on PacBio single-molecule sequencing technology with systematic identification of the AS events and APA sites as well as detection of differentially expressed transcripts among queen, drone, and worker bees. However, studies on the nanopore-sequencing-based full-length transcriptome of *A. mellifera* have been lacking until now.

The long reads generated by nanopore sequencing have been utilized in the refinement of reference genomes across multiple species, providing enhancements even for reference genomes for which chromosomal resolution has already been achieved [21,22,23,24,25]. For instance, Chen et al. employed full-length transcriptome data acquired via nanopore sequencing to refine the reference genome of *Nosema ceranae* [26]. This process resulted in the structural optimization of 2340 genes within the *N. ceranae* genome, featuring extensions at the 5′ end in 1182 genes and at the 3′ end in 1158 genes. In 2006, the *A. mellifera* genome (Amel_4.0) was first sequenced, revealing key genomic features; however, gene prediction was limited, indicating the need for improvement [27]. A subsequent version (Amel_4.5) published by Elisk et al. [28] in 2014, although more comprehensive, remained fragmented, with significant gaps in areas like centromeres and telomeres. In 2019, Wolberg et al. [29] enhanced the assembly to Amel_HAv3.1 using advanced sequencing techniques, achieving higher contiguity and structural integrity close to the chromosomal level. Nanopore sequencing is believed to offer an opportunity for improving the reference genome of *A. mellifera*.

In this current work, midgut samples of uninoculated and *N. ceranae*-inoculated *A. mellifera* workers were prepared and sequenced by nanopore sequencing technology, the full-length transcripts were identified followed by construction and annotation of the full-length transcriptome of *A. mellifera*. Additionally, detection, annotation, and verification of novel genes and transcripts were conducted, and the structures of those genes annotated in the *A. mellifera* reference genome were then optimized. Moreover, prediction and investigation of simple sequence repeats (SSRs), transcription factors (TFs), open reading frames (ORFs), and long non-coding RNAs (lncRNAs) were performed. In a follow-up study, the differential expression profile of the full-length transcripts in uninoculated and *N. ceranae*-inoculated *A. mellifera* workers and their potential functions will be investigated to decipher the host response to *N. ceranae* infection. Our data could not only enrich and improve the annotations of the current reference genome of *A. mellifera*, but also provide a solid basis for facilitating future molecular and -omics studies on *A. mellifera*.

## 2. Materials and Methods

### 2.1. Bee and Fungi

Three *A. mellifera* colonies were reared in the teaching apiary of the College of Bee Science and Biomedicine, Fujian Agriculture and Forestry University, Fuzhou, China. *N. ceranae* was previously prepared and conserved at the Honeybee Protection Laboratory of the College of Bee Science and Biomedicine, Fujian Agriculture and Forestry University, Fuzhou, China.

### 2.2. Fungal Inoculation and Midgut Sample Preparation

At 24 h after emergence, *A. mellifera* workers (*n* = 35) in the treatment group were each immobilized and fed 5 μL of 50% (*w*/*v*) sucrose solution containing 1 × 10^6^ *N. ceranae* spores, while workers (n = 35) in the control group were each immobilized and fed 5 μL of 50% (*w*/*v*) sucrose solution without spores. There was one cage each for the treatment and control groups. Workers in the cages were reared in two separate incubators at 34 ± 0.5 °C and 60%–70% RH. After initial feeding, both treatment and control groups were provided with a feeder containing 4 mL of 50% (*w*/*v*) sucrose solution without spores, which was replaced daily throughout the whole experiment. Each cage was carefully checked every 24 h, and the dead honeybees were removed each day. At 7 days post-inoculation (dpi) and 10 dpi, the midgut tissues of three workers in the treatment and control groups were dissected and transferred into clean Eppendorf (EP) tubes. The samples in the treatment and control groups collected at 7 dpi were named AmT1 and AmCK1, whereas the samples harvested at 10 dpi were named AmT2 and AmCK2, respectively. The midgut samples were quickly placed in liquid nitrogen and then kept in a −80 °C cryogenic refrigerator until the nanopore sequencing and molecular experiments were conducted.

### 2.3. Total RNA Extraction, cDNA Library Construction, and Nanopore Sequencing

The total RNA of midgut samples in the above-mentioned four groups were extracted using the TRizol Kit (Thermo Fisher Scientific, Bremen, Germany). Reverse transcription was then performed with a Maxima H Minus Reverse Transcriptase Kit (Thermo Fisher Scientific, Bremen, Germany). The genomic library for ONT sequencing was constructed using the ONT 1D ligation sequencing kit SQK-LSK109 (Oxford Nanopore Technologies, Oxford, UK) according to the manufacturer’s instructions. Full-length transcriptome sequencing of the constructed cDNA libraries was conducted on a PromethION sequencing platform (Oxford Nanopore Technologies, Oxford, UK). The duration of the sequencing reaction was 72 h. The nanopore-generated raw data were deposited in the NCBI SRA database (https://www.ncbi.nlm.nih.gov/sra/?term= (accessed on 19 April 2024)) and linked to the SRA number SUB14364771.

### 2.4. Data Quality Control and Full-Length Transcript Identification

Using the MINKNOW software (v. 1.4.3) local base caller, the sequencing data with original FAST5 format were converted to raw reads in FASTQ format. Next, all raw reads were filtered to remove low-quality (Q score < 7) and short raw reads (<500 bp). Based on the principle of nanopore cDNA sequencing, a primer sequence identified at both ends of a read was regarded as a full-length transcript sequence. The identified transcript sequences were aligned to the *N. ceranae* reference genome (assembly ASM98816v1), the aligned data were removed and the remaining data were subjected to subsequent analyses.

### 2.5. Annotation of Full-Length Transcripts

We combined the transcripts identified in the current research with those in the existing reference genome. This consolidated dataset was then aligned against the Nr (Non-redundant Protein Sequence) [30], SwissProt [31], KOG (eukaryotic Ortholog Groups) [32], eggNOG (Evolutionary Genealogy of Genes: Non-supervised Orthologous Groups) [33], Pfam (Protein family) [34], GO (Gene Ontology) [35], and KEGG (Kyoto Encyclopedia of Genes and Genomes) [36] databases using Diamond software (v2.0.15) to obtain corresponding annotations. The parameters for Diamond software were set as follows: -k 100 -e -evalue 1e-5 -f 5.

### 2.6. Identification and Annotation of Novel Transcripts and Novel Genes

We aligned the full-length transcripts identified in this study to the existing transcripts in the reference genome of *A. mellifera* (assembly Amel_HAv3.1) to identify novel transcripts and novel genes. Subsequently, these novel transcripts and novel genes were aligned to the Nr, Swiss-Prot, Pfam, KOG, eggNOG, GO, and KEGG databases to obtain the corresponding annotations.

### 2.7. Molecular Validation of Novel Transcripts

Specific upstream primers (F) and downstream primers (R) for three randomly selected novel transcripts (ONT.5166.8, ONT.6348.2, and ONT.6348.3) were designed utilizing PrimerPremierv5.0 software. The total RNA was isolated from the midgut tissues of uninoculated and *N. ceranae*-inoculated 8-day-old workers using the RNA-extraction kit (Plomag, Beijing, China), following which reverse transcription was conducted with a NeuScript II 1st strand cDNA synthesis kit (Nuoweizan, Nanjing, China). The obtained cDNA served as a template for RT-PCR amplification. The reaction was performed using the RT-PCR kit (Yisheng, Shanghai, China), with all procedures strictly adhering to the manufacturer’s instructions. The thermal-cycling conditions were as follows: an initial denaturation step at 94 °C for 5 min, followed by 30 cycles of denaturation at 94 °C for 30 s, annealing at 56 °C for 30 s, and extension at 72 °C for 10 min. The amplified products were detected by 1.8% agarose gel electrophoresis, and the target fragments were purified and then ligated to the pMD-19T vector (TaKaRa, Beijing, China), then transformed into *Escherichia coli* DH5α competent cells and identified by PCR. The bacteria liquid with a positive signal was subjected to Sanger sequencing by Sangon Biotech-Shanghai, China.

### 2.8. Structural Optimization of Annotated Genes in the A. mellifera Reference Genome

Gffcompare v0.12.7 software [37] was utilized to compare the identified transcripts in this study with the known transcripts annotated in the *A. mellifera* reference genome (Amel_HAv3.1). Following the comparison result, the annotated gene’s boundary was optimized by extending the upstream and (or) downstream untranslated region (UTR).

### 2.9. Prediction of SSR, ORF, TF Family, and LncRNA

The full-length transcripts longer than 500 bp were screened from the non-redundant full-length transcripts, and the SSR loci were then predicted using MISA v2.1 software (http://pgrc.ipk-gatersleben.de/misa/ (accessed on 3 February 2024)) with the de-fault parameters [37]. TransDecoder v5.7.1 software (https://github.com/TransDecoder/TransDecoder/wiki (accessed on 3 February 2024)) was employed to detect potential CDS and ORFs from all full-length transcripts, and those ORFs with both the start codon and stop codon were considered complete ORFs [38]. The sequences of predicted proteins from all full-length transcripts were aligned to the transcription factor (TF) database (transcription factor (TF) database) by hmmscan v2.41.2 (https://www.ebi.ac.uk/Tools/hmmer/search/hmmscan (accessed on 3 February 2024)) to obtain the predicted TF family. From the identified full-length transcripts, a combination of CPC [39], CNCI [40], CPAT [41], and Pfam Scan [42] was employed to predict lncRNAs, and the intersection was regarded with high confidence as a set of lncRNAs.

## 3. Results

### 3.1. Processing and Quality Control of Nanopore Sequencing Data

Here, nanopore sequencing of the AmCK1, AmCK2, AmT1, and AmT2 groups produced 7,100,161, 6,506,665, 5,942,745, and 6,664,923 raw reads, respectively, with N50 of 1347 bp, 1388 bp, 1328 bp, 1394 bp and average length of 1178 bp, 1201 bp, 1148 bp, 1196 bp (Table 1). The length distribution of raw reads ranged from 1 kb to more than 10 kb, with the largest group of raw reads distributed around 1 kb in length (Figure S1A–D). Additionally, the Q-value distribution of these raw reads was in the range Q6–Q16, with a significant number of raw reads exhibiting a quality value of Q9 (Figure S1E–H).

After quality control of raw reads, 6,928,170, 6,353,066, 5,745,048, and 6,416,987 clean reads were respectively identified in the aforementioned four groups, including 5,068,270 (73.15%), 4,857,960 (76.47%), 4,172,542 (72.63%) and 4,638,289 (72.28%) full-length clean reads (Table 2). The length distribution of the clean reads ranged from 1 kb to more than 10 kb, and the largest group consisted of reads 1 kb in length (Figure S2).

### 3.2. Identification of Full-Length Transcripts

After redundant full-length clean reads had been removed, 16,824, 17,708, 15,744, and 18,246 non-redundant full-length transcripts were detected in the four groups mentioned above, with N50 values of 1889 bp, 1830 bp, 1797 bp, and 1858 bp and average lengths of 1503 bp, 1478 bp, 1516 bp and 1546 bp, respectively (Table 3). Following the merger, a total of 28,019 non-redundant full-length clean reads were obtained. In addition, the length distribution of full-length transcripts was up to ~8 kb, with the greatest number of full-length transcripts distributed around 2 kb in length (Figure S3).

### 3.3. Annotation of the Full-Length Transcripts

**Figure 1:**
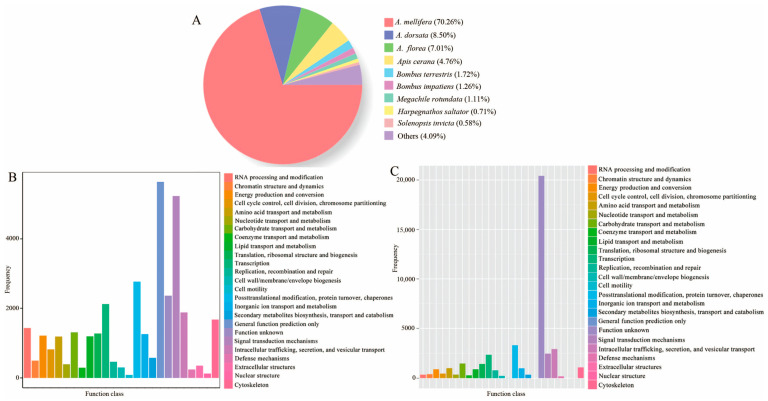
Annotations of *A. mellifera* full-length transcripts in the Nr (**A**), KOG (**B**), and eggNOG (**C**) databases.

Based on the union of transcripts identified in our study and those in the existing reference genome, a total of 43,666 full-length transcripts were successfully annotated to the Nr database. Among the annotated species, *A. mellifera* (30,678) had the greatest number of annotated full-length transcripts, followed by *Apis dorsata* (3711) and *Apis florea* (3059) (Table 4 and Table S1, Figure 1A). There were 30,945 full-length transcripts annotated to 25 functional categories in the KOG database. The top three categories were general function prediction (5642); signal-transduction mechanism (5236); and post-translational modifications, protein flipping and molecular chaperones (2767) (Table 4 and Table S1, Figure 1B). In addition, 41,771 full-length transcripts were annotated to 25 functional categories in the eggNOG database, including unknown function (20,417); post-translational modifications, protein flipping, and molecular chaperones (3300); and intracellular transport, assecretion, and vesicular transport (2923), as shown in Table 4 and Table S1, Figure 1C.

**Figure 2:**
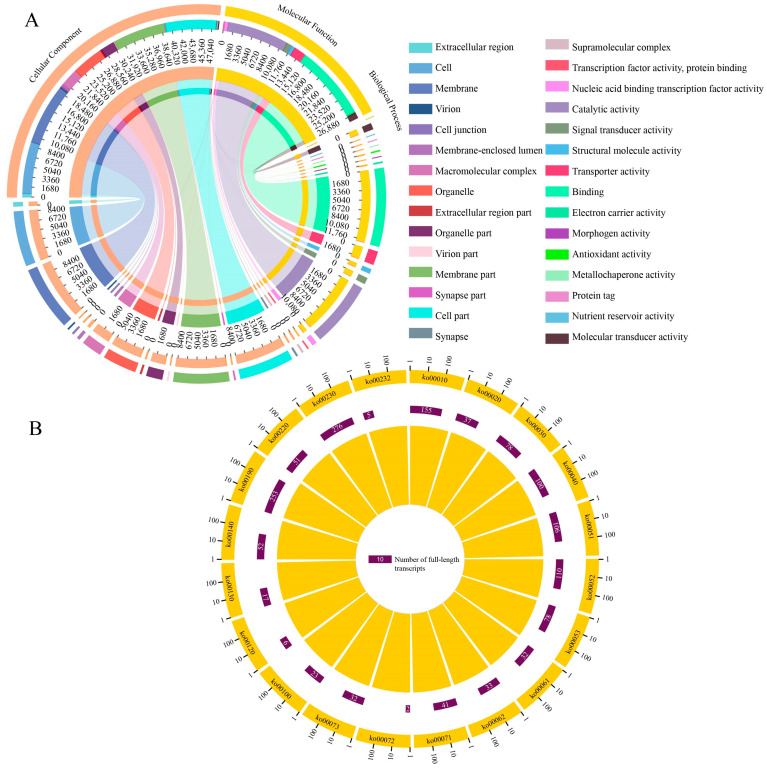
GO (**A**) and KEGG (**B**) database annotation of *A. mellifera* full-length transcripts.

In the GO database, 26,442 full-length transcripts were annotated to 53 functional terms, of which 16 were associated with cellular components such as the cell (8511) and membrane (9987), 15 were related to molecular functions such as catalytic activity (10,083) and transporter activity (2033), and 22 were relevant to biological processes such as cellular processes (10,391) and single-tissue processes (7121) (Table 4 and Table S1, Figure 2A). As presented in Table 4 and Table S1, Figure 2B, 24,532 full-length transcripts could be annotated to 231 KEGG pathways, including endocytosis (642), protein processing within the endoplasmic reticulum (589), carbon metabolism (527), ribonucleic acid transport (504), and oxidative phosphorylation (488).

### 3.4. Identification and Annotation of Novel Genes

In total, 501 novel genes were identified. In the Nr database, 255 novel genes could be annotated to *A. mellifera*, followed by *A. dorsata* (74) and *A. florea* (55) (Table 5 and Table S1, Figure S4A). In the KOG database, 193 novel genes could be annotated to 25 functional categories, such as signal-transduction mechanisms (32), general function prediction (31), and transcription (16) (Table 5 and Table S1, Figure S4B). As shown in Table 5 and Table S1, Figure S4C, 414 novel genes could be annotated to 25 functional categories in the eggNOG database, including unknown function (228), intracellular trafficking, secretion, and vesicular transport (31), as well as post-translational modification, protein folding, and chaperones (29). Additionally, 228 novel genes were annotated to 43 functional terms in the GO database, including 17 biological-process-related terms like metabolic process (69) and cellular process (69), 11 molecular-function-associated terms like catalytic activity (89) and transport activity (27), 15 cellular-component-related terms like membrane (96) and membrane component (81) (Table 5 and Table S1, Figure S4D). Moreover, 202 novel genes were annotated to 74 pathways in the KEGG database, such as oxidative phosphorylation (7), MAPK signaling pathway (7), protein processing in the endoplasmic reticulum (7), endocytosis (6), and sphingolipid metabolism (5) (Table 5 and Table S1, Figure S4E).

### 3.5. Identification, Annotation, and Validation of Novel Transcripts

**Figure 3:**
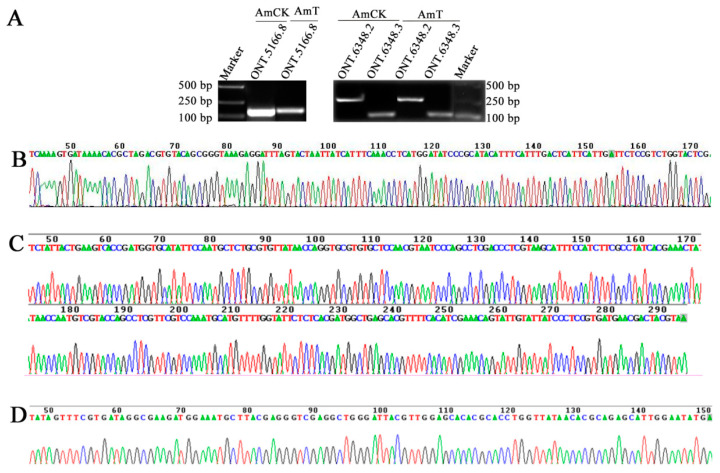
Molecular validation of novel transcripts in *A. mellifera*. (**A**) Agarose gel electrophoresis of the PCR-amplification products from ONT.5166.8, ONT.6348.2, and ONT.6348.3. (**B**–**D**) Peak diagrams of Sanger sequencing of the amplified fragments from ONT.5166.8, ONT.6348.2 and ONT.6348.3.

In total, 20,326 novel transcripts were identified; of these, 20,255 (Nr), 13,365 (KOG), 19,186 (egg-NOG), 12,093 (GO), and 11,703 (KEGG) were annotated (Figure S5, see also Table S1). RT-PCR results showed that fragments of the expected size were amplified from three randomly selected isoforms, including ONT.5166.8 (about 170 bp), ONT.6348.2 (about 290 bp), and ONT.6348.3 (about 150 bp) (Figure 3A). Additionally, the results of Sanger sequencing suggested that the sequences of these amplification fragments were consistent with those of predicted isoforms based on nanopore sequencing (Figure 3B–D). These results together verified the expression and sequences of these three isoforms, as well as the reliability of nanopore sequencing data.

### 3.6. Structural Optimization of Annotated Genes in the A. mellifera Reference Genome

Based on the identified genes, the structures of 4111 annotated genes in the *A. mellifera* reference genome were optimized. Among these, the 5′ UTRs of 2082 genes, the 3′ UTRs of 2029 genes, and both the 5′ and 3′ UTRs of 730 genes were extended (Table 6).

### 3.7. Identification of SSRs and Complete ORFs

**Figure 4:**
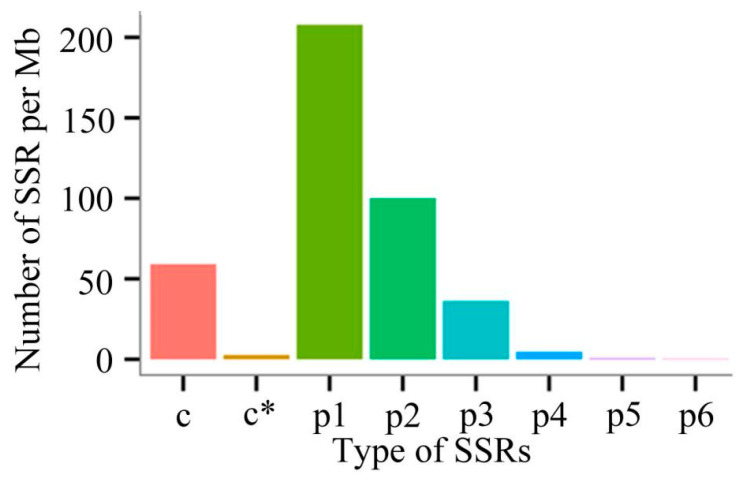
Density statistics of various types of SSRs. c: mixed SSRs containing at least two perfect SSRs at a distance less than 100 bp; c*: mixed SSRs with overlapping positions; p1: perfect single-base repeat, p2: perfect double-base repeat, p3: perfect three-base repeat, p4: perfect four-base repeat, p5: perfect five-base repeat, p6: perfect six-base repeat.

A total of 17,345 *A. mellifera* SSRs were identified. The quantities of mono-, di-, tri-, and tetra-nucleotide repeats were 8760, 4221, 1527, and 196, respectively (Table 7). Additionally, the density of mono-nucleotide repeats, di-nucleotide repeats, mixed SSRs, and tri-nucleotide repeats were 207.79/Mb, 100.12/Mb, 59.06/Mb, and 36.22/Mb, respectively (Figure 4).

**Figure 5:**
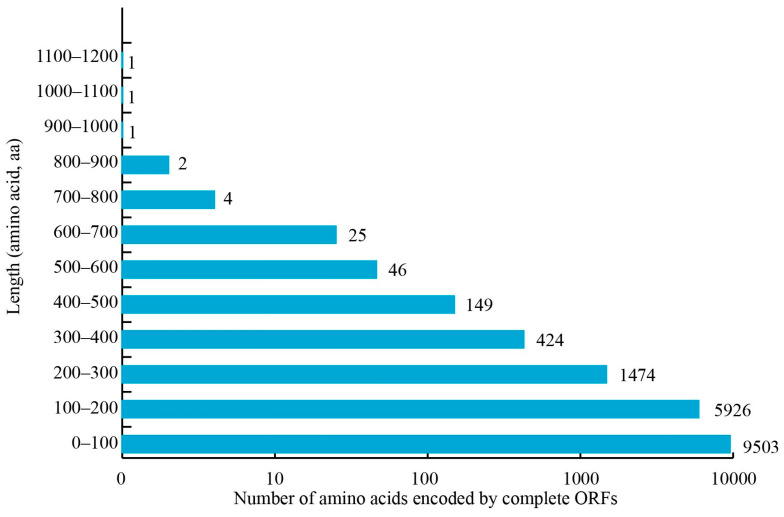
Length distribution of amino acids encoded by complete *A. mellifera* ORFs.

Based on the non-redundant transcripts identified in this study, 14,789 complete ORFs were predicted, with lengths up to 1200 aa (Figure 5). The most abundant ORFs were distributed in the range from 0 aa to 100 aa in length (54.13%), followed by those with length distributions of 100–200 aa (33.75%), 200–300 aa (8.40%), and 300–400 aa (2.42%) (Figure 5).

### 3.8. Identification of TF Families and lncRNAs

**Figure 6:**
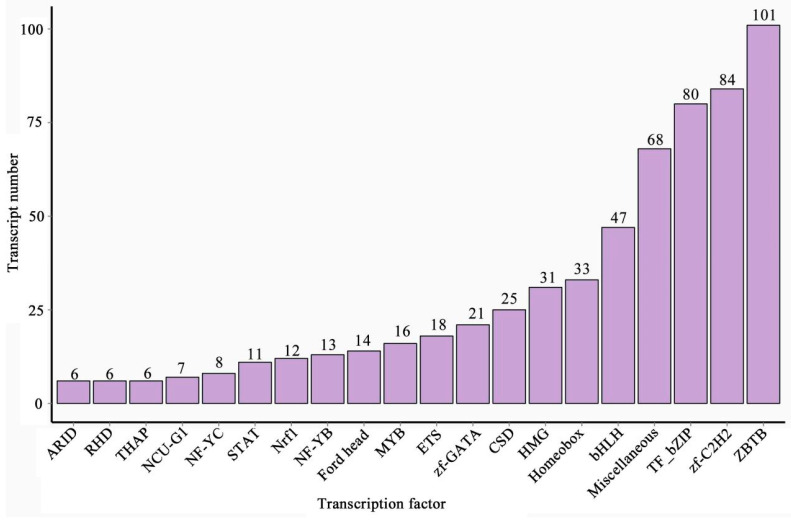
Counts of *A. mellifera* TF families and members. The number above each column indicates the quantity of involved members.

In total, 650 members within 37 TF families were predicted, and the top 10 TF families were ZBTB (101), zf-C2H2 (84), TF_bZIP (80), Miscellaneous (68), bHLH (47), Homeobox (33), HMG (31), CSD (25), zf-GATA (21), and ETS (18) (Figure 6).

**Figure 7:**
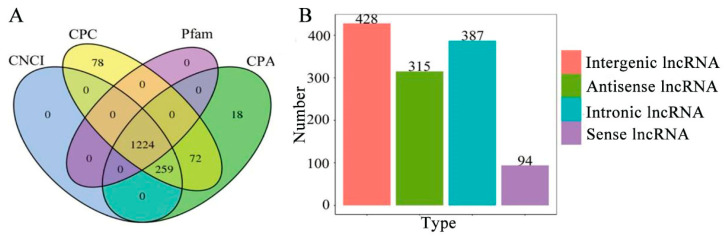
Number (**A**) and type (**B**) of *A*. *mellifera* lncRNAs. (**A**) Venn diagram of lncRNAs predicted by four software programs; (**B**) counts of various types of lncRNAs.

By using CNCI, CPC, Pfam, and CPAT, 1224 lncRNAs were finally identified (Figure 7A), including 428 intergenic lncRNAs, 387 intronic lncRNAs, 315 antisense lncRNAs, and 94 sense lncRNAs (Figure 7B).

## 4. Discussion

Here, based on long reads from nanopore sequencing of uninoculated and *N. ceranae*-inoculated workers’ midgut tissues, a total of 28,019 full-length transcripts were identified, with an N50 of 1876 bp and an average length of 1531 bp. Previously, following nanopore sequencing, the full-length transcriptomes of two widespread fungal pathogens, *N. ceranae* and *Ascosphaera apis*, were constructed by our group [43,44]. Recently, by using long reads produced by nanopore sequencing of cDNA libraries of larval guts, our team performed construction and annotation of the full-length transcriptome of the Asian honeybee, *Apis cerana*, including 40,562 full-length transcripts [45]. In this work, the midgut tissues of both uninoculated and *N. ceranae*-inoculated workers were subjected to nanopore sequencing. The reasons for this analysis were that the major objectives of this research were (1) to construct and annotate the first full-length transcriptome of *A*. *mellifera* and (2) to improve the annotation of current reference genome based on nanopore long reads. It is believed that a higher quality full-length transcriptome including more complete annotations could be constructed by using more data from nanopore sequencing of both uninoculated and *N. ceranae*-inoculated workers’ midguts. Our next work is to dissect the mechanism underlying the response of *A. mellifera* workers to *N. ceranae* invasion at the isoform level on basis of the high-quality long reads obtained in this study.

Notably, the number of full-length transcripts discovered in this work is more than the annotated transcripts in the *A. mellifera* reference genome (assembly Amel_HAv3.1), which was constructed using a subseries of latest sequencing technologies including PacBio, 10× Chromium, BioNano, and Hi-C [29]. This indicates that there is also room for improving the annotated transcripts in a chromosol-level genome utilizing Nanopore sequencing-produced long reads. Additionally, 43,712 (99.94%) full-length transcripts were found to be annotated to at least one of the above-mentioned five databases. However, as many as 25 (0.06%) full-length transcripts could not be annotated to any of these five databases, reflecting the necessity of continuous cloning and functional study of *A. mellifera* genes and isoforms. The constructed *A. mellifera* full-length transcriptome is a valuable resource for relevant molecular studies, such as the detection of genetic variants and cloning and functional investigation of various isoforms [46,47,48]. 

Nanopore-sequencing-produced long-read data have also been applied for optimizing the structures of annotated genes in the reference genomes of various animals, plants, and microorganisms [11,49,50]. In comparison with the genome of *A. mellifera* previously constructed using second-generation sequencing, the current reference genome of *A. mellifera* has a contig N50 of 5.381 Mbp and a scaffold N50 of 13.62 Mbp, representing a 120-fold improvement in contig-level contiguity and a 14-fold increase in scaffold-level contiguity [29]. On the basis of the full-length transcriptome data, we have optimized the annotated genes in the *A. mellifera* reference genome: the 5′ UTRs of 2082 existing genes have been extended, with extensions ranging from 1 bp to 162,043 bp, while the 3′ UTRs of 2059 existing genes have also been extended, with extensions spanning from 1 bp to 150,208 bp. In view of the close relationship between UTRs and regulation of gene expression in eukaryotes [51,52], the structural improvement of *A. mellifera* genes is of great importance for the cloning of full-length sequences of genes and the regulation of gene expression and transcription.

In recent years, nanopore sequencing has been employed to assemble high-quality genomes of diverse species like *Arabidopsis* [53], *Chrysomallon squamiferum* [54], and *Mycoplasma bovis* [55]. However, the current cost of nanopore-based genome sequencing is still high. In contrast, third-generation transcriptome sequencing is much more cost-effective. Accumulating evidence have shown that nanopore sequencing is highly efficient in exploring novel genes and transcripts [56,57]. Bayega et al. employed nanopore sequencing to elucidate transcription dynamics during early embryonic development in *Bactroceraoleae* and identified 1768 novel genes and 79,810 isoforms, significantly enhancing the transcriptome diversity [57]. Here, we discovered 501 novel genes and 20,326 novel transcripts, among which 489 (20,255), 193 (13,365), 414 (19,186), 228 (12,093), and 202 (11,703) novel genes (transcripts) could be annotated to the Nr, KOG, eggNOG, GO, and KEGG databases, respectively. These newly discovered genes and transcripts can further enrich the annotations in the *A. mellifera* reference genome. Additional work is needed to dissect the functions of these new genes and transcripts.

SSRs, which have several advantages such as simple experimental manipulation, good reproducibility, and high multi-allelicity, exhibit high levels of intraspecific and interspecific variation, making them useful for analysis of genetic diversity and genetic structure [16,58]. We previously identified 6312 *A. mellifera* SSRs by utilizing RNA-seq datasets from the gut tissues of worker larvae, among which the most abundant types were dinucleotide repeats (3435, 54.42%) and trinucleotide repeats (2051, 32.49%) [59]. Here, using nanopore sequencing data from the midgut tissues, 17,345 SSRs were identified, with the greatest number being single-nucleotide repeats (11,616, 67.0%). This suggests that greater quantities and more types of SSRs were detected using long reads generated from nanopore sequencing, a result similar to the findings in other animals [11,60,61]. These increased SSR resources can establish a solid foundation for future studies on the conservation and genetic breeding of *A. mellifera* [62]. Also, these SSRs will facilitate the interpretation of the genetic relationships among *A. mellifera* and their closely related species from the perspective of functional molecular markers [63,64,65].

TFs modulate the expression of target genes by binding to *cis*-acting elements within the promoter regions of these genes [59]. Previous studies have shown that TFs play important roles in insect physiological processes [63,64]. In *Drosophila*, the two TFs belonging to the ZBTB family, Chinmo and Broad, played antagonistic roles in the process of adult disc regeneration, affecting the self-renewal and regenerative potential of epithelial progenitor cells [66]. Here, 650 members of 37 TF families were identified, including 101 members of the ZBTB family, 84 members of the zf-C2H2 family, and 33 members of the Homeobox family, providing a valuable resource for continuous investigation of their functions in physiological and pathological processes in *A. mellifera.* LncRNAs are crucial regulators in diverse biological processes, ranging from gene expression [67] and chromatin regulation [68] to cellular development [69] and the stress response [70]. Studies based on second-generation sequencing have demonstrated that lncRNAs in *A. mellifera* were potentially engaged in transcriptional regulation, ovarian development, midgut growth, and the immune response [71]. Here, using nanopore sequencing data, 94 sense lncRNAs, 315 antisense lncRNAs, 387 intronic lncRNAs, and 428 intergenic lncRNAs were discovered, with most of these lncRNAs ranging from 250 nt to 5988 nt in length. Although fewer lncRNAs were discovered in this work (1224) than in our previous RNA-seq-based study (6353) [72], the average length was much longer. This offers an opportunity for cloning of the full lengths of these lncRNAs and investigation of their regulatory functions and action mechanisms.

## 5. Conclusions

This current work assembled and annotated the full-length transcriptome of *A. mellifera* using nanopore sequencing technology and refined the sequences and annotations of the *A. mellifera* reference genome through structural optimization of annotated genes as well as systematic identification of novel transcripts, TFs, and lncRNAs.

## Figures and Tables

**Table 1:** Summary of raw reads generated from nanopore sequencing.

cDNA Library	Sequence Number	Total base Number	N50 Length	Average Length	Maximum Length	Average Quality Value
AmCK1	7,100,161	8,368,331,508	1347	1178	13,936	Q10
AmCK2	6,506,665	7,816,378,025	1388	1201	31,074	Q10
AmT1	5,942,745	6,822,570,594	1328	1148	14,890	Q9
AmT2	6,664,923	7,976,689,232	1394	1196	16,430	Q9

**Table 2:** Overview of full-length clean reads.

cDNA Library	Number of Clean Reads	Number of Full-Length Clean Reads	Percentage of Full-Length Clean Reads
AmCK1	6,928,170	5,068,270	73.15%
AmCK2	6,353,066	4,857,960	76.47%
AmT1	5,745,048	4,172,542	72.63%
AmT2	6,416,987	4,638,289	72.28%

**Table 3:** Overview of *Apis mellifera* full-length transcripts.

cDNA Library	Sequence Numbers	Total Base Number	N50	Average Length	Maximum Length
AmCK1	16,824	25,303,104	1889	1503	6925
AmCK2	17,708	26,174,909	1830	1478	7525
AmT1	15,744	23,876,999	1797	1516	7556
AmT2	18,246	28,213,391	1858	1546	6130

**Table 4:** Overview of the annotation of full-length transcripts in *A. mellifera*. The figures enclosed in parentheses denote the number of annotated transcripts. Only the principal annotation details are shown.

Database	Annotation	
Nr (43,666)	*A*. *mellifera* (30,678)	
	*Apis dorsata* (3711)	
	*Apis florea* (3059)	
KOG (30,945)	General function prediction (5642)	
	Signal-transduction mechanism (5236)	
	Post-translational modifications, protein flipping and molecular chaperones (2767)	
eggNOG (41,771)	Unknown function (20,417)	
	Post-translational modifications, protein flipping, and molecular chaperones (3300)	
	Intracellular transport, assecretion, and vesicular transport (2923)	
GO (26,442)	Cellular components	Cell (8511)
		Membrane (9987)
	Molecular functions	Catalytic activity (10,083)
		Transporter activity (2033)
	Biological processes	Cellular processes (10,391)
		Single-tissue processes (7121)
KEGG (24,532)	Endocytosis (642)	
	Protein processing within the endoplasmic reticulum (589)	
	Carbon metabolism (527)	
	Ribonucleic acid transport (504)	
	Oxidative phosphorylation (488)	

**Table 5:** Overview of the annotation of novel genes in *A. mellifera*. The figures enclosed in parentheses denote the number of annotated novel genes. Only the principal annotation details are exhibited.

Database	Annotation	
Nr (489)	*A*. *mellifera* (255)	
	*A*. *dorsata* (74)	
	*A*. *florea* (55)	
KOG (193)	Signal-transduction mechanisms (32)	
	General function prediction (31)	
	Transcription (16)	
eggNOG (414)	Unknown function (228)	
	Intracellular trafficking, secretion, and vesicular transport (31)	
	Post-translational modification, protein folding, and chaperones (29)	
GO (228)	Cellular components	Membrane (96)
		Membrane component (81)
	Molecular functions	Catalytic activity (89)
		Transport activity (27)
	Biological processes	Metabolic process (69)
		Cellular process (69)
KEGG (202)	Oxidative phosphorylation (7)	
	MAPK signaling pathway (7)	
	Protein processing in the endoplasmic reticulum (7)	
	Endocytosis (6)	
	Sphingolipid metabolism (5)	

**Table 6:** Detailed information about structural optimization of annotated genes in the *A. mellifera* reference genome (10 presented only).

Gene ID	Gene Region	Strand	Termini	Original Location	Optimized Location
gene0	NC_037638.1:9269–12,174	−	5′	9273	9269
gene1	NC_037638.1:10,739–17,330	+	5′	10,792	10,739
gene1	NC_037638.1:10,739–17,330	+	3′	17,180	17,330
gene10000	NC_037649.1:10,681,873–10,685,795	+	3′	10,684,414	10,685,795
gene10002	NC_037649.1:10,690,083–10,692,393	−	5′	10,690,237	10,690,083
gene10003	NC_037649.1:10,692,186–10,694,102	+	5′	10,692,357	10,692,186
gene10003	NC_037649.1:10,692,186–10,694,102	+	3′	10,694,099	10,694,102
gene10008	NC_037649.1:10,709,808–10,712,252	−	5′	10,710,464	10,709,808
gene10009	NC_037649.1:10,712,599–10,715,281	+	3′	10,714,344	10,715,281
gene1001	NC_037638.1:15,188,580–15,189,776	−	5′	15,189,245	15,188,580

**Table 7:** Search results of *A. mellifera* SSRs based on MISA.

Search Items	Numbers
Number of sequences evaluated	26,201
Total base number of evaluated sequences (bp)	42,158,443
Total number of SSRs identified	20,680
Number of sequences containing SSRs	11,143
Number of sequences containing more than one SSR	4827
The number of SSRs in complex form	3335
Mononucleotide repeats	11,616
Dinucleotide repeat	6223
Trinucleotide repeat	2471
Tetranucleotide repeat	311
Pentanucleotide repeat	46
Hexanucleotide repeat	13

## Data Availability

All the data are contained within the article.

## Supplementary Materials

The following supporting information can be downloaded at: https://www.mdpi.com/article/10.3390/genes15060728/s1, Figure S1: Length and quality-value distribution of raw reads generated from nanopore sequencing; Figure S2: Length distribution of full-length clean reads; Figure S3: Overview of *Apis mellifera* full-length transcripts; Figure S4: Annotations of novel genes in *Apis mellifera* in the Nr (A), KOG (B), eggNOG (C), GO (D), and KEGG (E) databases; Figure S5: Annotations of novel transcripts of *Apis mellifera* in the Nr (A), KOG (B), eggNOG (C), GO (D), and KEGG (E) databases; Table S1: Functional-annotation information for all transcripts.

## References

[1] Klein A.M., Vaissière B.E., Cane J.H., Steffan-Dewenter I., Cunningham S.A., Kremen C., Tscharntke T. (2007). Importance of Pollinators in Changing Landscapes for World Crops. Proc. Biol. Sci..

[2] Genersch E. (2010). Honey Bee Pathology: Current Threats to Honey Bees and Beekeeping. Appl. Microbiol. Biotechnol..

[3] Han F., Wallberg A., Webster M.T. (2012). From Where Did the Western Honeybee (*Apis mellifera*) Originate?. Ecol. Evol..

[4] Zeng Z.J. (2017). Apiculture.

[5] Fuentes-Pardo A.P., Ruzzante D.E. (2017). Whole-Genome Sequencing Approaches for Conservation Biology: Advantages, Limitations and Practical Recommendations. Mol. Ecol..

[6] Eisenstein M. (2019). Playing a Long Game. Nat. Methods.

[7] Fang L., Guo L., Zhang M., Li X., Deng Z. (2022). Analysis of Polyadenylation Signal Usage with Full-Length Transcriptome in *Spodoptera frugiperda* (Lepidoptera: Noctuidae). Insects.

[8] Zhao X., Li C., Zhang H., Yan C., Sun Q., Wang J., Yuan C., Shan S. (2020). Alternative Splicing Profiling Provides Insights into the Molecular Mechanisms of Peanut Peg Development. BMC Plant Biol..

[9] de Klerk E., den Dunnen J.T., ‘t Hoen P.A.C. (2014). RNA Sequencing: From Tag-Based Profiling to Resolving Complete Transcript Structure. Cell. Mol. Life Sci..

[10] Byrne A., Cole C., Volden R., Vollmers C. (2019). Realizing the Potential of Full-Length Transcriptome Sequencing. Philos. Trans. R. Soc. B Biol. Sci..

[11] Lin J., Guan L., Ge L., Liu G., Bai Y., Liu X. (2021). Nanopore-Based Full-Length Transcriptome Sequencing of Muscovy Duck (*Cairina moschata*) Ovary. Poult. Sci..

[12] Zhang X., Han C., Gao H., Cao Y. (2019). Comparative Transcriptome Analysis of the Garden Aspzaragus (*Asparagus officinalis* L.) Reveals the Molecular Mechanism for Growth with Arbuscular Mycorrhizal Fungi under Salinity Stress. Plant Physiol. Biochem..

[13] Jenjaroenpun P., Wongsurawat T., Pereira R., Patumcharoenpol P., Ussery D.W., Nielsen J., Nookaew I. (2018). Complete Genomic and Transcriptional Landscape Analysis Using Third-Generation Sequencing: A Case Study of Saccharomyces Cerevisiae CEN.PK113-7D. Nucleic Acids Res..

[14] Xing L., Wu Q., Xi Y., Huang C., Liu W., Wan F., Qian W. (2022). Full-Length Codling Moth Transcriptome Atlas Revealed by Single-Molecule Real-Time Sequencing. Genomics.

[15] Ouyang H., Wang X., Zheng X., Lu W., Qin F., Chen C. (2021). Full-Length SMRT Transcriptome Sequencing and SSR Analysis of *Bactrocera dorsalis* (Hendel). Insects.

[16] Sun X., Li H. (2023). Full-Length Transcriptome Combined with RNA Sequence Analysis of *Fraxinus chinensis*. Genes Genom..

[17] Bovo S., Ribani A., Utzeri V.J., Taurisano V., Schiavo G., Bolner M., Fontanesi L. (2021). Application of Next Generation Semiconductor-Based Sequencing for the Identification of *Apis mellifera* Complementary Sex Determiner (csd) Alleles from Honey DNA. Insects.

[18] Manfredini F., Brown M.J., Vergoz V., Oldroyd B.P. (2015). RNA-sequencing elucidates the regulation of behavioural transitions associated with the mating process in honey bee queens. BMC Genom..

[19] Doublet V., Poeschl Y., Gogol-Döring A., Alaux C., Annoscia D., Aurori C., Barribeau S.M., Bedoya-Reina O.C., Brown M.J., Bull J.C. (2017). Unity in defence: Honeybee workers exhibit conserved molecular responses to diverse pathogens. BMC Genom..

[20] Zheng S.Y., Pan L.X., Cheng F.P., Jin M.J., Wang Z.L. (2023). A Global Survey of the Full-Length Transcriptome of *Apis mellifera* by Single-Molecule Long-Read Sequencing. Int. J. Mol. Sci..

[21] Lee Y.G., Choi S.C., Kang Y., Kim K.M., Kang C.S., Kim C. (2019). Constructing a Reference Genome in a Single Lab: The Possibility to Use Oxford Nanopore Technology. Plants.

[22] Salson M., Orjuela J., Mariac C., Zekraouï L., Couderc M., Arribat S., Rodde N., Faye A., Kane N.A., Tranchant-Dubreuil C. (2023). An Improved Assembly of the Pearl Millet Reference Genome Using Oxford Nanopore Long Reads and Optical Mapping. G3.

[23] Rousseau-Gueutin M., Belser C., Da Silva C., Richard G., Istace B., Cruaud C., Falentin C., Boideau F., Boutte J., Delourme R. (2020). Long-Read Assembly of the *Brassica napus* Reference Genome Darmor-Bzh. Gigascience.

[24] Pham G.M., Hamilton J.P., Wood J.C., Burke J.T., Zhao H., Vaillancourt B., Ou S., Jiang J., Buell C.R. (2020). Construction of a Chromosome-Scale Long-Read Reference Genome Assembly for Potato. Gigascience.

[25] Cuenca-Guardiola J., de la Morena-Barrio B., García J.L., Sanchis-Juan A., Corral J., Fernández-Breis J.T. (2023). Improvement of Large Copy Number Variant Detection by Whole Genome Nanopore Sequencing. J. Adv. Res..

[26] Chen H., Fan Y., Jiang H., Wang J., Fan X., Zhu Z., Long Q., Cai Z., Zhen Y., Fu Z. (2021). Improvement of *Nosema ceranae* Genome Annotation Based on Nanopore Full-Length Transcriptome Data. Sci. Agric. Sin..

[27] (2006). Honeybee Genome Sequencing Consortium Insights into Social Insects from the Genome of the Honeybee *Apis mellifera*. Nature.

[28] Elsik C.G., Worley K.C., Bennett A.K., Beye M., Camara F., Childers C.P., de Graaf D.C., Debyser G., Deng J., Devreese B. (2014). Finding the Missing Honey Bee Genes: Lessons Learned from a Genome Upgrade. BMC Genom..

[29] Wallberg A., Bunikis I., Pettersson O.V., Mosbech M.-B., Childers A.K., Evans J.D., Mikheyev A.S., Robertson H.M., Robinson G.E., Webster M.T. (2019). A Hybrid de Novo Genome Assembly of the Honeybee, *Apis mellifera*, with Chromosome-Length Scaffolds. BMC Genom..

[30] Deng Y., Li J., Wu S., Zhu Y., Chen Y., He F. (2006). Integrated Nr Database in Protein Annotation System and its Localization. Comput. Eng..

[31] UniProt Consortium T. (2018). UniProt: The Universal Protein Knowledgebase. Nucleic Acids Res..

[32] Koonin E.V., Fedorova N.D., Jackson J.D., Jacobs A.R., Krylov D.M., Makarova K.S., Mazumder R., Mekhedov S.L., Nikolskaya A.N., Rao B.S. (2004). A Comprehensive Evolutionary Classification of Proteins Encoded in Complete Eukaryotic Genomes. Genome Biol..

[33] Powell S., Forslund K., Szklarczyk D., Trachana K., Roth A., Huerta-Cepas J., Gabaldón T., Rattei T., Creevey C., Kuhn M. (2014). eggNOG v4.0: Nested Orthology Inference Across 3686 Organisms. Nucleic Acids Res..

[34] Kanehisa M., Goto S., Kawashima S., Okuno Y., Hattori M. (2004). The KEGG Resource for Deciphering the Genome. Nucleic Acids Res..

[35] Ashburner M., Ball C.A., Blake J.A., Botstein D., Butler H., Cherry J.M., Davis A.P., Dolinski K., Dwight S.S., Eppig J.T. (2000). Gene Ontology: Tool for the Unification of Biology. The Gene Ontology Consortium. Nat. Genet..

[36] McKenna A., Hanna M., Banks E., Sivachenko A., Cibulskis K., Kernytsky A., Garimella K., Altshuler D., Gabriel S., Daly M. (2010). The Genome Analysis Toolkit: A MapReduce Framework for Analyzing next-Generation DNA Sequencing Data. Genome Res..

[37] Thiel T., Michalek W., Varshney R.K., Graner A. (2003). Exploiting EST Databases for the Development and Characterization of Gene-Derived SSR-Markers in Barley (*Hordeum vulgare* L.). Theor. Appl. Genet..

[38] Du Y., Fu Z.M., Zhu Z.W., Wang J., Feng R.R., Wang X.N., Jiang H.B., Fan Y.C., Fan X.X., Xiong C.L. (2020). Elongation of genic untranslated regions, exploration of SSR loci and identification of unannotated genes and transcripts based on the nanopore sequencing dataset of *Ascosphaera apis*. Acta Entomol. Sin..

[39] Kong L., Zhang Y., Ye Z., Liu X., Zhao S., Wei L., Gao G. (2007). CPC: Assess the Protein-Coding Potential of Transcripts Using Sequence Features and Support Vector Machine. Nucleic Acids Res..

[40] Sun L., Luo H., Bu D., Zhao G., Yu K., Zhang C., Liu Y., Chen R., Zhao Y. (2013). Utilizing Sequence Intrinsic Composition to Classify Protein-Coding and Long Non-Coding Transcripts. Nucleic Acids Res..

[41] Wang L., Park H.J., Dasari S., Wang S., Kocher J.-P., Li W. (2013). CPAT: Coding-Potential Assessment Tool Using an Alignment-Free Logistic Regression Model. Nucleic Acids Res..

[42] Mistry J., Chuguransky S., Williams L., Qureshi M., Salazar G.A., Sonnhammer E.L.L., Tosatto S.C.E., Paladin L., Raj S., Richardson L.J. (2021). Pfam: The protein families database in 2021. Nucleic Acids Res..

[43] Chen H., Du Y., Fan X., Zhu Z., Jiang H., Wang J., Fan Y., Xiong C., Zheng Y., Fu Z. (2021). Construction and annotation of the full-length transcriptome of *Nosema ceranae* based on the third-generation nanopore sequencing technology. Acta Entomol. Sin..

[44] Du Y., Zhu Z., Wang J., Wang X., Jiang H., Fan Y., Fan X., Chen H., Long Q., Cai Z. (2021). Construction and Annotation of *Ascosphaera apis* Full-Length Transcriptome Utilizing Nanopore Third-Generation Long-Read Sequencing Technology. Sci. Agric. Sin..

[45] Song Y., Li K., Zang H., Jin X., Fan X., Zou P., Chen D., Fu Z., Guo R. (2024). Construction and annotation of the full-length transcriptome of the larval gut of *Apis cerana cerana* (Hymenoptera: Apidae) worker. Acta Entomol. Sin..

[46] Lin B., Hui J., Mao H. (2021). Nanopore Technology and Its Applications in Gene Sequencing. Biosensors.

[47] Leger A., Amaral P.P., Pandolfini L., Capitanchik C., Capraro F., Miano V., Migliori V., Toolan-Kerr P., Sideri T., Enright A.J. (2021). RNA Modifications Detection by Comparative Nanopore direct RNA Sequencing. Nat. Commun..

[48] Hotaling S., Wilcox E.R., Heckenhauer J., Stewart R.J., Frandsen P.B. (2023). Highly Accurate Long Reads are Crucial for Realizing the Potential of Biodiversity Genomics. BMC Genom..

[49] Grünberger F., Ferreira-Cerca S., Grohmann D. (2022). Nanopore Sequencing of RNA and cDNA Molecules in *Escherichia coli*. RNA.

[50] Zhao L., Zhang H., Kohnen M.V., Prasad K., Gu L., Reddy A.S.N. (2019). Analysis of Transcriptome and Epitranscriptome in Plants Using PacBio Iso-Seq and Nanopore-based Direct RNA Sequencing. Front. Genet..

[51] Liu H., Yin J., Xiao M., Gao C., Mason A.S., Zhao Z., Liu Y., Li J., Fu D. (2012). Characterization and Evolution of 5′ and 3′ Untranslated Regions in Eukaryotes. Gene.

[52] Srivastava A.K., Lu Y., Zinta G., Lang Z., Zhu J.K. (2018). UTR-Dependent Control of Gene Expression in Plants. Trends Plant Sci..

[53] Cui J., Shen N., Lu Z., Xu G., Wang Y., Jin B. (2020). Analysis and Comprehensive Comparison of PacBio and Nanopore-based RNA Sequencing of the *Arabidopsis* Transcriptome. Plant Methods.

[54] Sun J., Li R., Chen C., Sigwart J.D., Kocot K.M. (2021). Benchmarking Oxford Nanopore Read Assemblers for High-Quality Molluscan Genomes. Philos. Trans. R. Soc. B Biol. Sci..

[55] Vereecke N., Bokma J., Haesebrouck F., Nauwynck H., Boyen F., Pardon B., Theuns S. (2020). High Quality Genome Assemblies of *Mycoplasma bovis* Using a Taxon-specific Bonito Basecaller for MinION and Flongle Long-read Nanopore sequencing. BMC Bioinform..

[56] Glinos D.A., Garborcauskas G., Hoffman P., Ehsan N., Jiang L., Gokden A., Dai X., Aguet F., Brown K.L., Garimella K. (2022). Transcriptome Variation in Human Tissues Revealed by Long-read Sequencing. Nature.

[57] Bayega A., Oikonomopoulos S., Gregoriou M.E., Tsoumani K.T., Giakountis A., Wang Y.C., Mathiopoulos K.D., Ragoussis J. (2021). Nanopore Long-read RNA-seq and Absolute Quantification Delineate Transcription Dynamics in Early Embryo Development of an Insect Pest. Sci. Rep..

[58] Buschiazzo E., Gemmell N.J. (2006). The Rise, Fall and Renaissance of Microsatellites in Eukaryotic Genomes. Bioessays.

[59] Guo R., Chen H., Zhuang T., Xiong C., Zheng Y., Fu Z., Chen H., Chen D. (2018). Exploitation of SSR markers for *Apis mellifera* ligustica based on transcriptome data. J. Anhui Agric. Univ..

[60] Gaikwad A.B., Kumari R., Yadav S., Rangan P., Wankhede D.P., Bhat K.V. (2023). Small Cardamom Genome: Development and Utilization of Microsatellite Markers from a Draft Genome Sequence of *Elettaria cardamomum* Maton. Front. Plant Sci..

[61] Gurjar M.S., Kumar T.P.J., Shakouka M.A., Saharan M.S., Rawat L., Aggarwal R. (2023). Draft Genome Sequencing of *Tilletia caries* Inciting Common Bunt of Wheat Provides Pathogenicity-related Genes. Front. Microbiol..

[62] Liu F., Guo Q.S., Shi H.Z., Cheng B.X., Lu Y.X., Gou L., Wang J., Shen W.B., Yan S.M., Wu M.J. (2016). Genetic Variation in *Whitmania pigra*, *Hirudo nipponica* and *Poecilobdella manillensis*, Three Endemic and Endangered Species in China Using SSR and TRAP Markers. Gene.

[63] Lim S., Lee J., Lee H.J., Park K.H., Kim D.S., Min S.R., Jang W.S., Kim T.I., Kim H. (2017). The genetic diversity among strawberry breeding resources based on SSRs. Sci. Agric..

[64] Al-Shammari A.M.A., Hamdi G.J., Al-Mahdawi M.A.S., Mohammed N.K. (2021). Genetic diversity analysis and DNA fingerprinting of tomato breeding lines using SSR markers. Agraarteadus J. Agric. Sci..

[65] Jing S., Liu B., Peng L., Peng X., Zhu L., Fu Q., He G. (2012). Development and use of EST-SSR markers for assessing genetic diversity in the brown planthopper (*Nilaparvata lugens* Stål). Bull. Entomol. Res..

[66] Narbonne-Reveau K., Maurange C. (2019). Developmental Regulation of Regenerative Potential in Drosophila by Ecdysone through a Bistable Loop of ZBTB Transcription Factors. PLoS Biol..

[67] Gil N., Ulitsky I. (2020). Regulation of Gene Expression by Cis-acting Long Non-coding RNAs. Nat. Rev. Genet..

[68] Man H.J., Marsden P.A. (2019). LncRNAs and Epigenetic Regulation of Vascular Endothelium: Genome Positioning System and Regulators of Chromatin Modifiers. Curr. Opin. Pharmacol..

[69] Schmitz S.U., Grote P., Herrmann B.G. (2016). Mechanisms of Long Noncoding RNA Function in Development and Disease. Cell. Mol. Life Sci..

[70] Vourc’h C., Dufour S., Timcheva K., Seigneurin-Berny D., Verdel A. (2022). HSF1-Activated Non-Coding Stress Response: Satellite lncRNAs and Beyond, an Emerging Story with a Complex Scenario. Genes.

[71] Wang J., Sun M., Long Q., Fan Y., Wu Y., Guo Y., Zhang K., Shi C., Chen D., Guo R. (2022). Analysis of Highly-Expressed LncRNAs Function in Regulating Midgut Development of *Apis mellifera ligustica* worker. J. Sichuan Univ. (Nat. Sci. Ed.).

[72] Chen D., Chen H., Du Y., Zhou D., Geng S., Wang H., Wan J., Xiong C., Zheng Y., Guo R. (2019). Genome-Wide Identification of Long Non-Coding RNAs and Their Regulatory Networks Involved in *Apis mellifera ligustica* Response to *Nosema ceranae* Infection. Insects.

