# Supplementary material for: Construction of a Full-Length Transcriptome of Western Honeybee Midgut Tissue and Improved Genome Annotation
Source: Genes (Basel). 2024 Jun 1;15(6):728. doi: 10.3390/genes15060728 (PMC11202838; doi:10.3390/genes15060728)
Supplement: Supplementary file 1 [file genes-15-00728-s001.zip › Figure S1-S5.pdf]

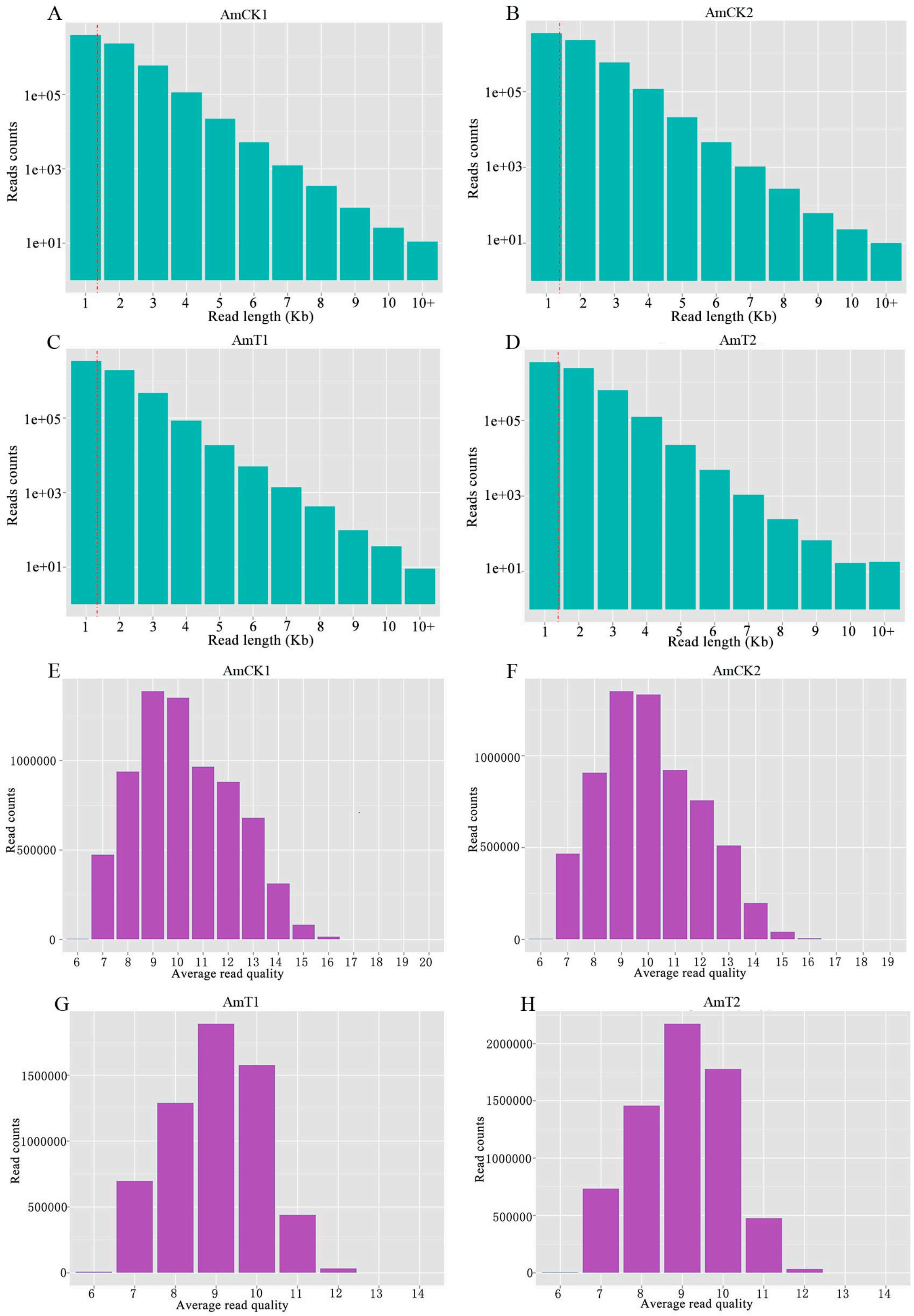

Figure S1 Length and quality value distribution of raw reads generated from Nanopore sequencing. A-D: Length distribution of raw reads; E-H: Quality value of raw reads

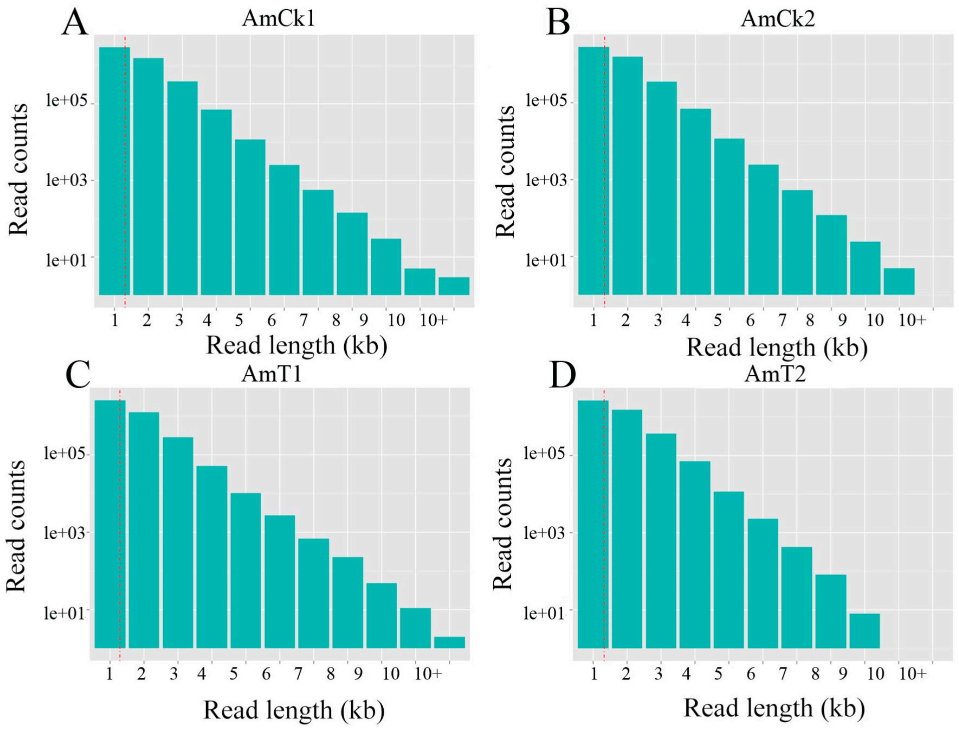

Figure S2. Length distribution of full-length clean reads.

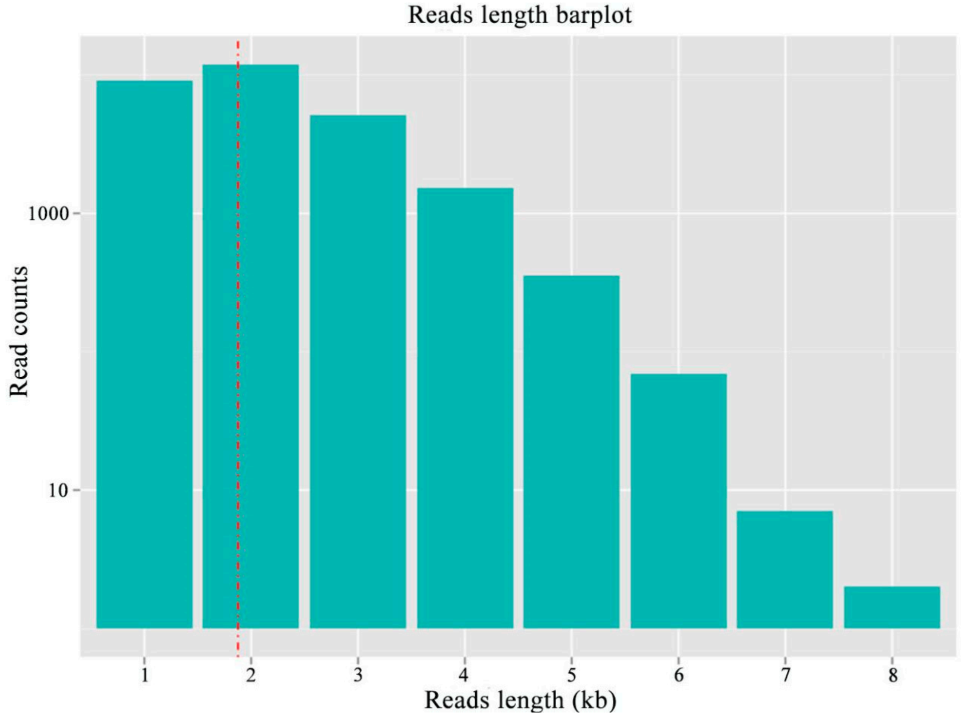

Figure S3 Overview of *Apis mellifera* full-length transcripts.

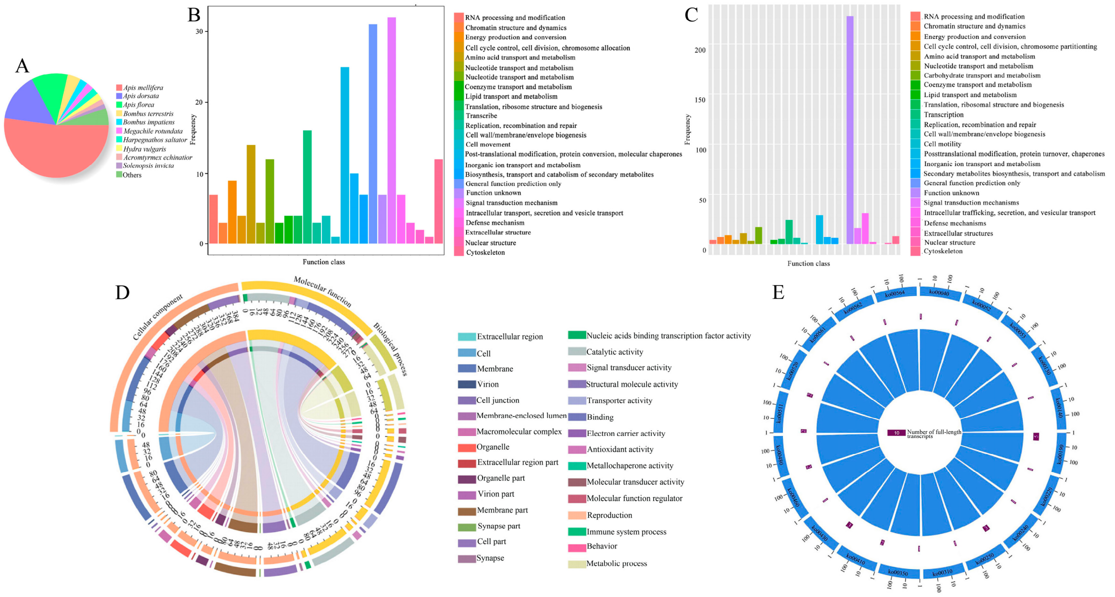

Figure S4 Annotations of novel genes in *Apis mellifera* in Nr (A), KOG (B), eggNOG (C), GO(D), and KEGG(E) database.

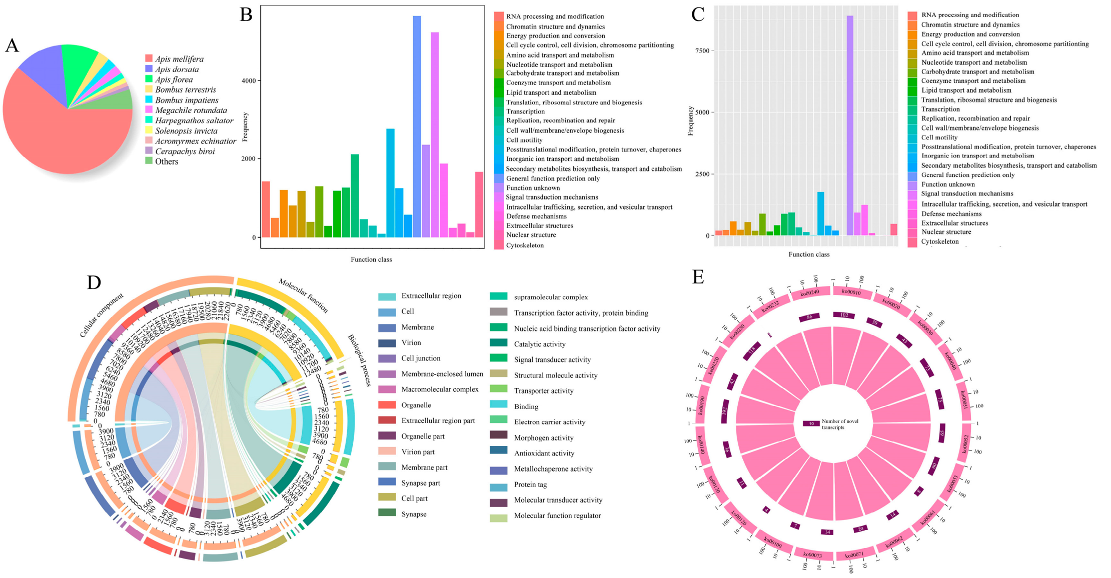

Figure S5 Annotations of novel transcripts of *Apis mellifera* in Nr (A), KOG (B), eggNOG (C), GO(D), and KEGG(E) database.
